# Oro-Gustatory Perception of Dietary Lipids and Calcium Signaling in Taste Bud Cells Are Altered in Nutritionally Obesity-Prone *Psammomys obesus*

**DOI:** 10.1371/journal.pone.0068532

**Published:** 2013-08-01

**Authors:** Souleymane Abdoul-Azize, Feriel Atek-Mebarki, Arezki Bitam, Hassimi Sadou, Elhadj Ahmed Koceïr, Naim Akhtar Khan

**Affiliations:** 1 Physiologie de la Nutrition & Toxicologie, UMR U866 INSERM/Université de Bourgogne/Agro-Sup, Dijon, France; 2 Bioenergetics and Intermediary Metabolism Laboratory, FSB, University of Sciences and Technology Houari Boumédiene (USTHB), Algiers, Algeria; 3 Laboratoire de Nutrition, Université Abdou Moumouni, Niamey, Niger; Aligarh Muslim University, India

## Abstract

Since the increasing prevalence of obesity is one of the major health problems of the modern era, understanding the mechanisms of oro-gustatory detection of dietary fat is critical for the prevention and treatment of obesity. We have conducted the present study on *Psammomys obesus*, the rodent desert gerbil which is a unique polygenic natural animal model of obesity. Our results show that obese animals exhibit a strong preference for lipid solutions in a two-bottle test. Interestingly, the expression of CD36, a lipido-receptor, in taste buds cells (TBC), isolated from circumvallate papillae, was decreased at mRNA level, but remained unaltered at protein level, in obese animals. We further studied the effects of linoleic acid (LA), a long-chain fatty acid, on the increases in free intracellular calcium (Ca^2+^) concentrations, [Ca^2+^]i, in the TBC of *P. obesus*. LA induced increases in [Ca^2+^]i, largely *via* CD36, from intracellular pool, followed by the opening of store-operated Ca^2+^ (SOC) channels in the TBC of these animals. The action of this fatty acid on the increases in [Ca^2+^]i was higher in obese animals than that in controls. However, the release of Ca^2+^ from intracellular stores, studied also by employing thapsigargin, was lower in TBC of obese animals than control rodents. In this study, we show, for the first time, that increased lipid intake and altered Ca^2+^ signaling in TBC are associated with obesity in *Psammomys obesus*.

## Introduction

According to the World Health Organization (WHO) [1], obesity has reached epidemic proportions in the world, with over 1.6 billion adults being overweight and, at least, 500 million being clinically obese. One out of ten adults worldwide is obese, having important consequences in terms of public health. Obesity, particularly visceral obesity, is a risk factor for several diseases such as type 2 diabetes, cardiovascular complications, hypertension and some types of cancers [2].

It is generally assumed that excessive intake of dietary fat contributes to weight gain and obesity. There are several factors which influence fat consumption, including energy density and palatability [3]. Besides, oral taste sensitivity to fatty acids may influence food ingestion and, consequently, regulate body weight. Lanfer *et al.* [4] have conducted a study on 1696 children aged 6–9 years in different countries of the European Union and concluded that fat preference is related to body weight status. Stewart *et al.* [5] have classified obese subjects as hypo- or hyper-sensitive to oleic acid taste detection and concluded that hyposensitive subjects consumed significantly more energy (fat, saturated fat, fatty foods) and had greater body mass index (BMI). The hyposensitive subjects were less perceptive of small changes in the fat contents compared to hypersensitive subjects. In another study, Stewart and Keast [6] reported that in lean subjects, the consumption of the high-fat diet significantly decreased taste sensitivity to oleic acid.

The lipid-binding glycoprotein CD36, expressed on circumvallate papillae (CVP) of mouse and rat tongue, has been implicated in oro-gustatory perception of dietary lipids [7], [8], [9]. Recent data from our team show that fatty acid-induced Ca^2+^ signaling in CD36-positive taste bud cells, regulated by stromal interaction molecule 1 (STIM1) *via* CD36, is implicated in spontaneous preference for fat [9]. The importance of CD36 can be further supported with the study of Pepino *et al.* [10] who have shown that a single nucleotide polymorphism (SNP) of CD36, leading to its inactivation, resulted in a decreased oral lipid taste perception in obese subjects. However, the intracellular mechanisms involved in decreased lipid taste sensitivity in taste bud cells (TBC) remain to be explored.

Several rodent models have been used to investigate the pathogenesis of obesity but they do not reflect the human disease sufficiently. *Psammomys obesus*, a desert gerbil, is a unique polygenic animal model for obesity and type 2 diabetes [11]. These rodents remain lean and free from diabetes in their native desert habitat, subsisting on a hypocaloric diet composed mainly of halophilic plants [12]. When transferred to the laboratory and allowed free access to standard rodent chow, metabolic disturbances such as obesity, hyperglycemia and dyslipidemia occur in *P. obesus* relative to their lean littermates [13], [14], [15]. Diabetes development is very fast in these rodents. The animals reach the irreversible hypoinsulinemic stage of the disease, in which a marked reduction of β-cell mass is apparent, within 4–6 weeks of high caloric diet [11]. Obesity development in *P. obesus*, accompanied by low liver glucose-6-phosphate hydrolysis, contributes to insulin resistant state, with a high lipogenic activity [16]. Besides, we have recently demonstrated that obese *P. obesus* showed a hepatic deterioration which was accompanied by enhanced oxidative stress, further contributing to deleterious outcomes of insulin resistance [12]. Hyperglycemia in these animals is reversible, except for the hypoinsulinemic end stage of the disease; normoglycemia could be obtained by limiting the caloric intake [17]. However, the relationships between natural obesity and oro-sensory detection of dietary lipids in *P. obesus* are not yet known and deserve deep investigation.

Keeping in view the afore-mentioned arguments, it was thought worthwhile to assess the impact of obesity on lipid taste perception and calcium signaling in CD36-positive taste bud cells (TBC) isolated from circumvallate papillae of *P. obesus*.

## Materials and Methods

### Chemicals

The culture medium RPMI 1640 and L-glutamine were purchased from Lonza Verviers SPRL (Verviers, Belgium). Fura-2/AM was procured from Invitrogen (Invitrogen, CA, USA). Elastase and dispase were purchased from Serlabo (Bonneuil/Marne, France) and Roche Diagnostics (Paris, France), respectively. Anti-CD36 antibody coupled to phycoerythrin and anti-α-gustducin antibody were procured from Santa Cruz Biotechnology Inc. (USA). Anti-CD36 antibody, used for western blots, was procured from R & D (UK). Sulfo-N-succinimidyl-oleate (SSO) was a generous gift from JF Glatz (Maastricht, The Netherlands). All other chemicals including linoleic acid (18∶2 n-6), collagenase type-I, and trypsin inhibitor were obtained from Sigma Chemicals (St. Louis, MO, USA).

### Animals and diet

The rodents (*Psammomys obesus)* were trapped in the area of Beni-Abbes (30°7′ North latitude and 2°10′ West longitude) in Algerian West Sahara and transported to Algiers. When the sand rats were captured, they were subjected to acclimatization in the animal house from 15 to 30 days. The animals were maintained in suitable cages under controlled temperature and light conditions. The animals were identified for the sex. The age of male gerbils, used in our study, was approximatively from 2 to 3 months, based on the body weight. The weighing is the main selection criteria in most of the studies on these animals. The animals were weighed and, at the beginning of the experiments, their body weights were 72±5 g. Each group consisted of 10 animals. The gerbils of control group were maintained on *Salsola foetida* throughout the experimentation. The animals of obese group were progressively given the laboratory diet and, after a period of 4 weeks, they were completely maintained on it until the duration of the experimentation, *i.e.,* 18 weeks.

For control animals, we chose the desert plant *Salsola foetida*, a low caloric diet, as these animals feed this plant in their natural habitat and they do not develop obesity [18]. As compared to the plants of chenopodiacae family, the laboratory diet is hypercaloric. If we compare 1 g of *Salsola foetida* with 1 g of laboratory diet, Salsola would furnish with 0.4 Kcal/g of fresh plant compared to the labortory chow which would provide with 3.25 Kcal/g. The laboratory diet contained the following: proteins 25%, lipids 7.5%, carbohydrates 47.4%, humidity/water 9%, fibers (cellulose) 4%, minerals 7.1%. The *Salsola foetida* contained the following: proteins 3.53%, lipids 0.4%, carbohydrates: 8.42%, humidity/water 80.79%, fibers (cellulose) 5.97%, minerals 6.86% [19]. Food and water were supplied *ad libitum* except for the taste preference tests (see here-after).

All experimental procedures were approved by the Algerian Institutional Animal Care Committee which belongs National Administration of Algerian Higher Education and Scientific Research (Algiers). The study was a part of a bilateral Franco-Algerian collaborative project “Tassili” (grant number:12MDU855). The authorization to capture the animals in desert region was given by the Ministry of Higher Education, Algeria. The animals were sacrificed by cervical dislocation.

### Biochemical analysis

Each animal was monitored for body weight, blood glucose and insulin. For biochemical analysis, fasted animals were killed by cervical decapitation at the end of treatments, without anesthesia to avoid any further stress, and blood samples were collected in tubes containing heparin. Plasma glucose and lipids fractions were measured by a spectrophotometric method adapted on a Cobas Mira automatic analyser. Plasma immunoreactive insulin was estimated by the Phadebas insulin test. Rat insulin (Novo) was used as standard.

### Hepatic lipids assays

Extraction of hepatic lipids (glycerides, cholesterol, fatty acids) was carried out according to Folch *et al.* [20], as described elsewhere [21]. Total lipids were measured gravimetrically. Total cholesterol and cholesterol fractions (HDL, LDL) were evaluated by the kit (Boehringer) as per instructions furnished with. Phospholipids were determined colorimetrically [22].

### Two-bottle preference test

The experiments on the spontaneous preference for lipid-enriched solutions were performed by means of two-bottle preference test. The animals first accustomed to water drinking in two bottles for a period of 24 hrs. The next day, the animals were subjected, during the diurnal period, to two bottles: one control and one test bottle, both containing 0.3% xanthan gum (Sigma Aldrich) homogenized in water. In the test bottle, 1% of colza oil was added. The xanthan gum was used to emulsify the oil and to minimize textural cues between the two solutions. To avoid the development of side preferences, positions of the bottles were changed during each experiment. After 12 hours, consumption of each solution was analyzed by weighing the bottles and preference for the experimental solution was estimated by calculating the ratio between the consumption of the experimental solution and the total consumption (in grams). We also assessed whether obese gerbils exhibit an altered sweet preference by subjecting them, similarly, to two bottle-test: one with water and another with 4% sucrose (w/v).

### Isolation of CD36-positive taste bud cells (TBC) from circumvallate papillae (CVP)

The CD36-positive cells from lingual CVP of *Psammomys obesus* were isolated according to our previously published procedure [23], [24]. Briefly, lingual epithelium was separated from connective tissues by enzymatic dissociation (elastase and dispase mixture, 2 mg/ml each, in Tyrode buffer: 120 mM NaCl; 5 mM KCl; 10 mM HEPES; 1 mM CaCl_2_; 1 mM MgCl_2_; 10 mM glucose; 10 mM Na^+^ pyruvate, pH 7.4). CD36-positive cells were isolated by incubating lingual epithelium in RPMI 1640 medium containing 2 mM EDTA, 1.2 mg/ml elastase, 0.6 mg/ml collagenase (type I), and 0.6 mg/ml trypsin inhibitor at 37°C for 10 minutes, followed by centrifugation (600 g, 10 minutes). The mixture of different cell populations was incubated with anti-CD36 antibody coupled to phycoerythrin for 2 hours, followed by a wash with PBS, pH 7.4 (600 g, 10 minutes), and resuspended in a solution containing microbeads coupled to anti-phycoerythrin IgG. The CD36-positive TBC were isolated by passing through the MACS columns of the Miltenyi magnet system. Both the cell populations, after separation, were suspended in fresh RPMI 1640 medium containing 10% fetal calf serum, 200 U/ml penicillin, and 0.2 mg/ml streptomycin, seeded onto a BD BioCoat Poly-D-Lysine-coated dishes, and cultured for 24 hours. At the end of this period, the cells were used for the experiments or stained with trypan blue to assess their viability.

### Detection of mRNA by real-time PCR (RT-PCR)

Total RNA was extracted from CD36-positive TBC by using TRIzol (Invitrogen) and subjected to DNase treatment using the RNAse-free DNAse Set (QIAGEN). One microgram of total RNA was reverse transcribed with SuperScript II RNAse H reverse transcriptase using oligo(dT) according to the manufacturer's instructions (Invitrogen). RT-PCR was performed on the iCycler iQ real-time detection system, and amplification was undertaken by using SYBR Green I detection. Primers against the genes of interest were designed using the available mRNA sequence information in NCBI Genbank. Basic local alignment search tool (Blast, NCBI, available at http://www.ncbi.nlm.nih.gov/blast.cgi) was used for the verification of primers. Because the genes studied have not yet been sequenced for *P. obesus*, we designed primers based on the conserved regions in mRNA of the three well-characterized species: *Homosapiens, Rattus norvegicus, and Mus musculus* [25]. The sequences of the genes used are as follows: β-Actin (Forward: 5′-AGAGGGAAATCGTGCGTGAC-3′, Reverse: 5′-CAATAGTGATGACCTGGCCGT-3′); CD36 (Forward: 5′- ACTCTCTCCTCGGATGGCTAGCTG -3′, Reverse: 5′- CCACAGTTCCGATCGCAGCCC -3′); α-gustducin (Forward 5′-GTTGGCTGAAATAAT TAAACG-3′, Reverse: 5′-ATCTCTGGCCACCTACATC-3′). The amplification was carried out in a total volume of 25 μl, which contained 12.5 μl SYBR Green Supermix buffer (50 mM KCl; 20 mM Tris-HCl [pH 8.4]; 3 mM MgCl_2_; 0.2 mM of each dNTP, 0.63 U iTaq DNA polymerase, and SYBR Green 1.0 nM fluorescein) and 12.5 μl (0.3 μM) of each primer and diluted cDNA. Results were evaluated by iCycler iQ software and relative quantification of mRNA in different groups was determined.

### Western blot detection of CD36 in TBC

The CD36-positive TBC, isolated from lingual CVP of control and obese animals, were lysed with 50 µl of buffer containing the following: 20 mM, HEPES pH 7.3; 1 mM, EDTA; 1 mM, EGTA; 0.15 mM, NaCl; 1%, Triton X-100; 10% glycerol; 1 mM, phenylmethylsulfonyl fluoride (PMSF); 2 mM, sodium orthovanadate and anti-protease cocktail (2 µl in 1 ml of buffer). After centrifugation (13 000 *g*×1 min), cells lysates were used immediately or stored at −20°C. The protein content was determined by bicinchoninic acid (Pierce, France). Denatured proteins (30 µg) were separated by SDS-PAGE (10%) and transferred to polyvinylidine difluoride (PVDF) membranes, and immunodetection was performed by using anti-CD36 antibodies (1∶1000 dilution). After treating the membranes with peroxidase-conjugated anti-goat secondary antibodies, peroxidase activity was detected with ECL reagents (Amersham, France).

### Measurement of Ca^2+^ signaling

The CD36-positive TBC (2×10^6^/ml) were cultured on WillCodish wells with a glass bottom and loaded with Fura-2/AM (1 µM) for 60 min at 37°C in loading buffer which contained the following: 110 mM NaCl; 5.4 mM KCl; 25 mM NaHCO_3_; 0.8 mM MgCl_2_; 0.4 mM KH_2_PO_4_; 20 mM Hepes-Na; 0.33 mM Na_2_HPO_4_; 1.2 mM CaCl_2_, pH 7.4. After loading, the cells were washed three times and remained suspended in the identical buffer. The changes in intracellular Ca^2+^ (*F*_340_/*F*_380_) were monitored under the Nikon microscope (TiU) by using S-fluor 40× oil immersion objective. The planes were taken at *Z* intervals of 0.3 μm, and NIS-Elements software was used to deconvolve the images. The microscope was equipped with EM-CCD (Lucas) camera for real time recording of 16-bit digital images. The dual excitation fluorescence imaging system was used for studies of individual cells. The changes in [Ca^2+^]i were expressed as ΔRatio, which was calculated as the difference between the peak *F*_340_/*F*_380_ ratios. The data were summarized from the large number of individual cells (20–40 cells in a single run, with 3–9 identical experiments done in at least three cell preparations). For experiments in Ca^2+^-free medium, CaCl_2_ was replaced by EGTA (2 mM).

### Statistical analysis

Statistical analysis of data was carried out using Statistica (version 4.1, Statsoft, Paris, France). Data are presented as means ± SEM. The significance of the differences between mean values was determined by analysis of variance one way, followed by a least-significant-difference (LSD) test. For all the tests, the significance level chosen was p<0.05.

## Results

### Obesity is associated with increased spontaneous preference for lipids in *P. obesus*

The gerbils fed a high caloric chow developed metabolic syndrome like features, with an increase in their body weight (Table 1). The levels of plasma lipids, particularly triglyceridemia, were higher in obese animals than control rodents (Table 1). Insulinemia and glycemia were also significantly higher in obese animals than control animals (Table 1).

**Table 1:** Biochemical analysis of plasma and liver parameters in *Psammomys Obesus*.

Parameters	Control group	Obese group	*p*-values
Body weight (g)	79.76±4.50	121±30	*p*<0.001
Insulin (µU/ml)	23.50±3.20	150±19	*p*<0.001
Glucose (g/l)	0.58±0.10	0.81±0.17	*p*<0.01
Total Cholesterol (g/l)	0.58±0.37	1.01±0.35	*p*<0.001
HDL cholesterol (g/l)	0.41±0.04	0.55±0.03	*p*<0.01
LDL cholesterol (g/l)	0.37±0.02	0.72±0.03	*p*<0.001
Triglycerides (g/l)	0.52±0.39	1.48±0.06	*p*<0.001
Phospholipids (g/l)	0.71±0.22	0.72±0.18	*p*<0.05
Liver mass/body weight (%)	3.39±0.09	4.15±0.80	*p*<0.05
Hepatic total lipids (g/100g wet wt)	3.99±0.14	4.36±0.60	*p*<0.01
Hepatic total cholesterol (g/100g wet wt)	0.27±0.01	0.289±0.07	*p*<0.05
Hepatic total glycerides (g/100g wet wt)	0.29±0.01	0.446±0.02	*p*<0.001

Values are means ± SEM, n = 10.

Our data show that the liver size increases with the development of obesity in *Psammomys obesus*. This increase in hepatic mass is positively correlated with hyperinsulinemia, insulin resistance, hypertriglyceridemia and hepatic triglycerides. In this investigation, we also measured the hepatic mass-body weight ratio which was increased in obese animals (Table 1).

To explore whether the preference for lipids might be affected by obesity, the two-bottle preference test was performed in control and obese animals. We observed that the obese sand rats exhibited a high preference for an oily solution (Table-2). The control lean animals did not drink any of the solutions. The obese gerbils also exhibited an increased taste preference for a sweet solution (Table-2).

**Table 2:** Spontaneous preference of lipid and sweet solutions in *Psammomys Obesus*.

Intake (g/12 hrs/animal)	Control group	Obese group
Water (xanthane)	0.00	0.12±0.01
Colza oil (1%, v/v)	0.00	7.55±0.06*
Sucrose (4%, w/v)	0.88±0.7	16.5±1.30*

Values are means ± SEM, n = 10. The asterisk (*p<0.001) shows the significant differences in obese group animals, drank a solution containing colza oil or sucrose and compared to those that drank water (xanthane).

### CD36 expression is altered in TBC of obese *P. obesus*

**Figure 1:**
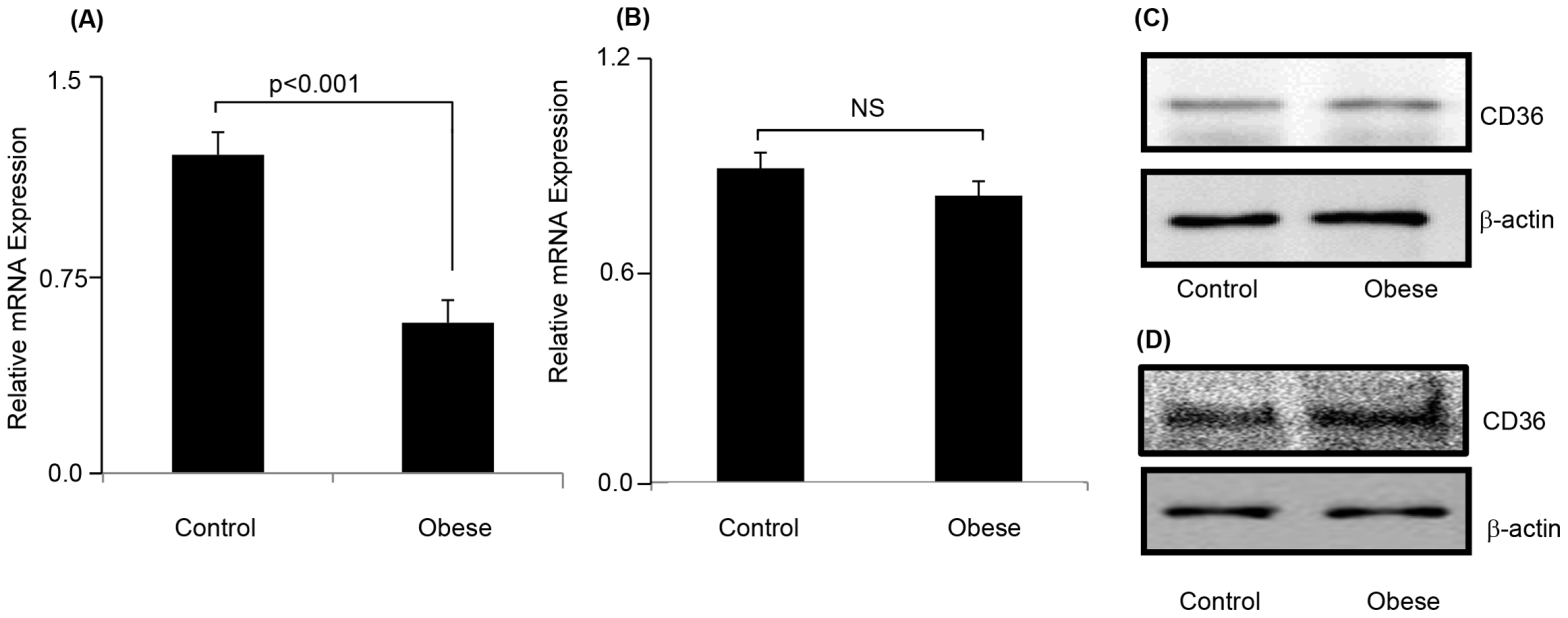
Expression of and CD36 and α-gustducin. The CD36-positive TBC were isolated from CVP of *P. obesus* as described in Materials and Methods. Relative mRNA expression of CD36 (Fig. 1A) and α-gustducin (Fig. 1B) in CD36-positive TBC cells was assessed. p<0.001 shows the significant differences between TBC of obese and control animals. NS = insignificant differences. The CD36 and β-actin proteins were also detected by western blots in CD36-positive TBC (Fig. 1C) and intestinal cells (Fig. 1D) of control and obese animals. For experimental details, see Materials and Methods.

The CD36 has been detected in various tissues including the lingual epithelium [26], and is thought to be a lipido-receptor, implicated in the oro-sensory detection of dietary lipids [7], [27]. Therefore, we detected the expression of CD36 mRNA and protein contents in TBC. Figure 1A shows that there was lesser expression of CD36 mRNA in TBC of obese animals than that in controls. The expression of CD36 protein was not altered in TBC (Fig. 1C) and intestinal tissues (Fig. 1D) between control and obese gerbils.

We were also interested in determining the mRNA expression of α-gustducin, a G-protein considered to be a specific marker of taste receptor cells, in control and obese Psammomys. We observed no difference in the expression of α-gustducin mRNA between control and obese animals (Fig. 1B), showing that the integrity of taste receptor cells (TRC) is not affected in TBC of obese animals.

### Linoleic acid-mediated Ca^2+^ signaling is altered in TBC of obese *P. obesus*

**Figure 2:**
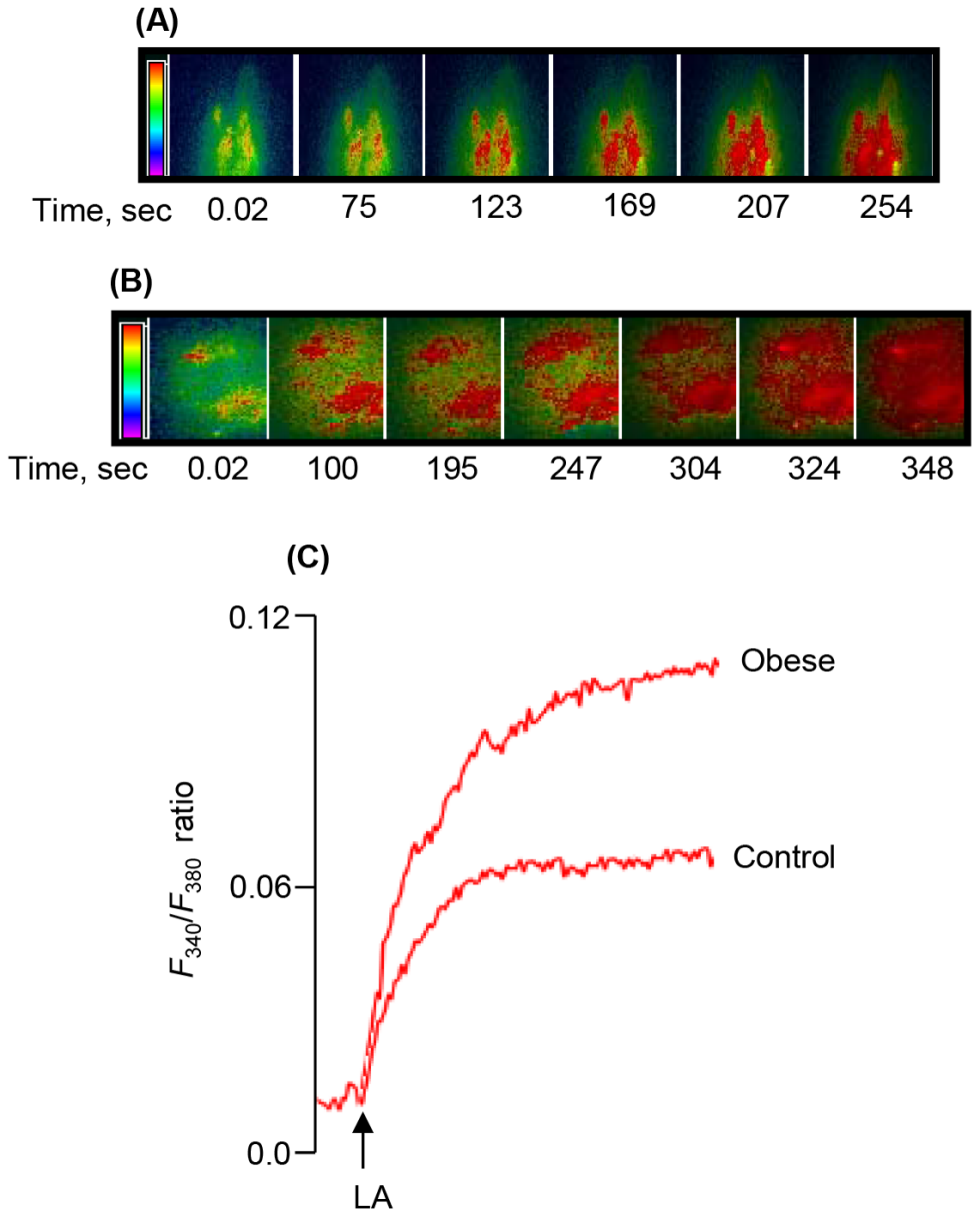
Effects of LA on the increases in [Ca^2+^]i in CD36-positive TBC. The CD36-positive TBC were isolated from CVP of *P. obesus* as described in Materials and Methods. Ca^2+^ imaging studies were performed on CD36-positive TBC in calcium-containing media. The changes in intracellular Ca^2+^ (F_340_/F_380_) were monitored under the Nikon microscope (TiU) by using S-fluor 40x oil immersion objectives, as described in Materials and Methods. Colored time-lapse changes show the kinetics of the rise in [Ca^2+^]i, following addition of linoleic acid (LA), in a CD36-positive taste bud cell, freshly isolated from CVP of control (A) and obese (B) animals. (C) represents the single traces of observations and the arrow indicates when the test molecule (LA, 20 µM) was added into the cuvette without interruptions in the recording.

**Figure 3:**
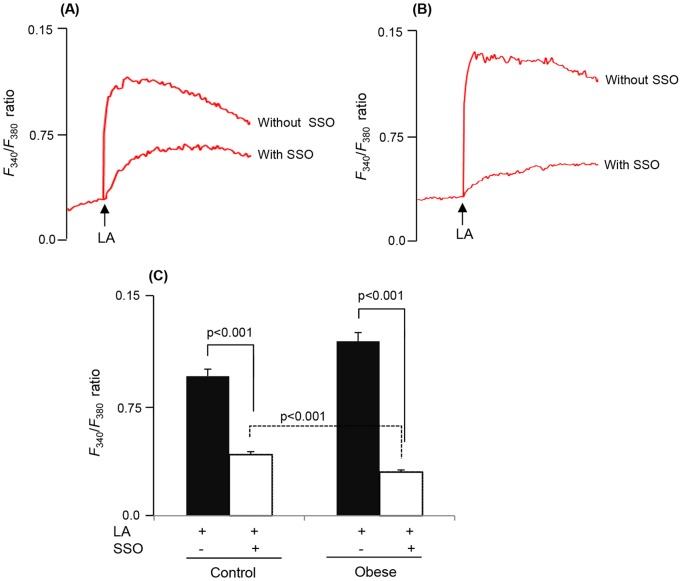
LA-induced increases in [Ca^2+^]i *via* CD36 in TBC. The CD36-positive TBC cells were incubated with or without SSO (50 µM) for 20 min and then treated with LA at 20 µM. Ca^2+^ imaging studies were performed on CD36-positive TBC in Ca^2+^-containing media. The changes in intracellular Ca^2+^ (F_340_/F_380_) were monitored under the Nikon microscope (TiU) by using S-fluor 40x oil immersion objectives, as described in Materials and Methods. The arrows indicate when the test molecule (LA, 20 µM) was added into the cuvette without interruptions in the recording in TBC of control (A) and obese (B) animals. (C) represents histograms of the changes in [Ca^2+^]i.

We observed that LA, a long-chain fatty acid (LCFA), induced a rapid rise in [Ca^2+^]i in CD36-positive TBC (Fig. 2A, B, C). The LA-induced increases in [Ca^2+^]i were higher in TBC of obese animals than those in control rodents (Fig. 2C). Furthermore, we employed sulfo-*N*-succinimidyl-oleate (SSO), the CD36 inhibitor [28], which significantly diminished Ca^2+^ signaling in TBC of control and obese animals (Fig. 3A, B, C). However, the action of SSO was more pronounced in TBC of obese animals than that in controls (Fig. 3C).

**Figure 4:**
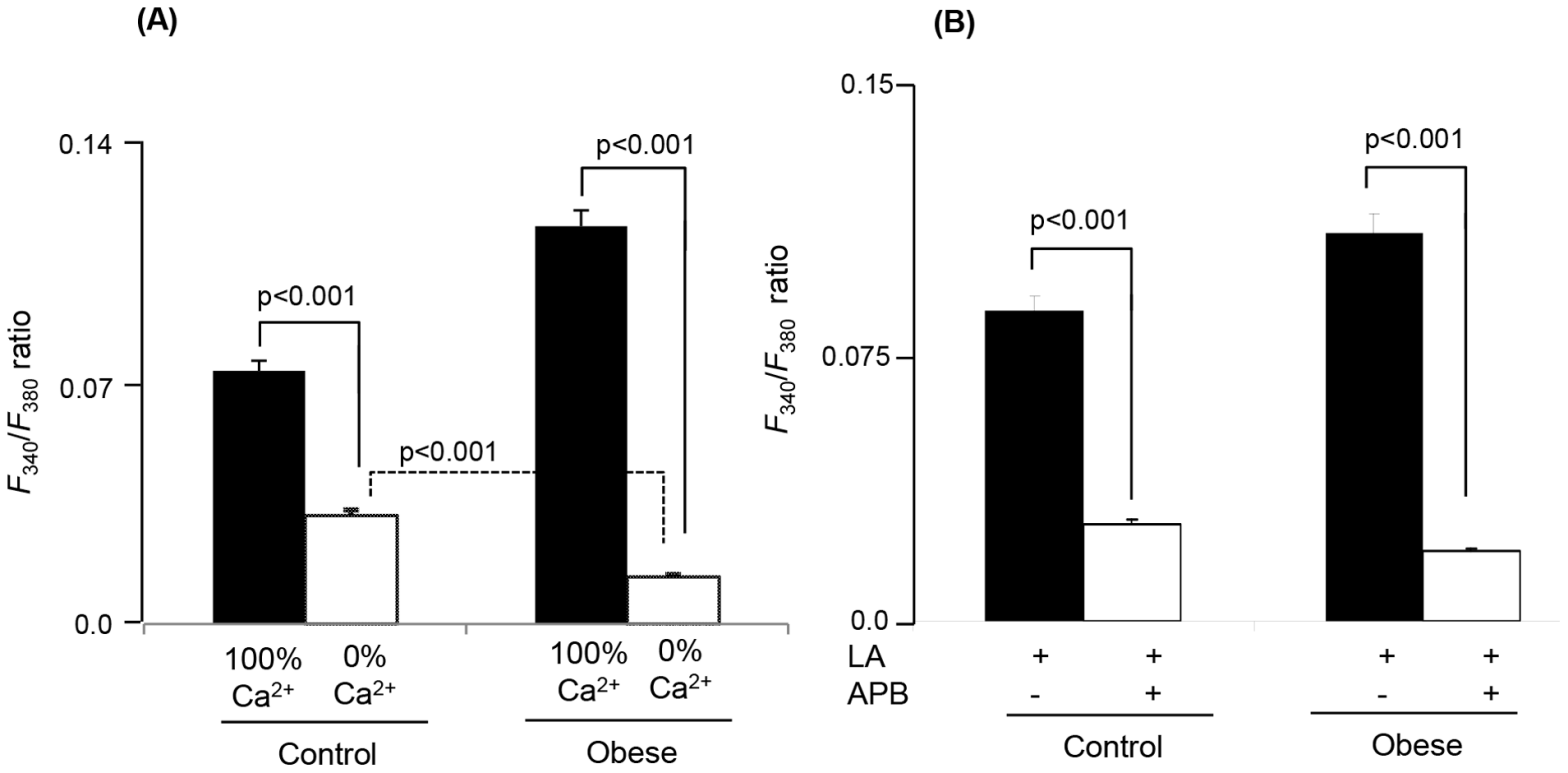
Implication of Ca^2+^ influx in LA-induced increases in [Ca^2+^]i in CD36-positive TBC. The CD36-positive TBC were isolated from CVP of *P. obesus* as described in Materials and Methods. Ca^2+^ imaging studies were performed in Ca^2+^-free (0% Ca^2+^) or Ca^2+^-containing (100% Ca^2+^) media. The changes in intracellular Ca^2+^ (F_340_/F_380_) were monitored under the Nikon microscope (TiU) by using S-fluor 40× oil immersion objectives. (A) represents the experiments performed in Ca^2+^-containing (100% Ca^2+^) and Ca^2+^-free medium (0% Ca^2+^). (B) represents the Δ increases in [Ca^2+^]i in the presence of a SOC channel blocker (ABP at 5 µM) in Ca^2+^-containing (100% Ca^2+^) medium. In both the experimental conditions, LA was used at 20 µM.

In order to assess the implication of intracellular Ca^2+^, we replaced Ca^2+^ from extracellular medium by EGTA, hence, termed as 0% Ca^2+^. Figure 4A shows that replacement of Ca^2+^ by EGTA significantly decreased the increases in [Ca^2+^]i in TBC of control and obese sand rats; however, the decrease in Ca^2+^ signaling was more pronounced in TBC of obese animals than that in controls. Whether LA induces the opening of store-operated Ca^2+^ (SOC) channels in 100% Ca^2+^ medium, we employed 2-aminoethoxydiphenyl borate (APB), a blocker of these channels. Figure 4B shows that prior-addition of APB into the cuvette significantly decreased Ca^2+^ signaling in TBC of control and obese animals.

### Thapsigargin-mediated Ca^2+^ signaling is altered in TBC of obese *P. obesus*

Thapsigargin (TG) is the most widely used inhibitor of the ubiquitous sarcoplasmic-endoplasmic Ca^2+^-ATPase (SERCA) in mammalian cells. Over the past ten years, this guaianolide compound of plant origin has become a popular tool in most of studies directed at elucidating the implication of Ca^2+^ that belongs to endoplasmic reticulum, ER [29]. TG deprives ER of its Ca^2+^ refilling process and, therefore, induces increases in [Ca^2+^]i.

**Figure 5:**
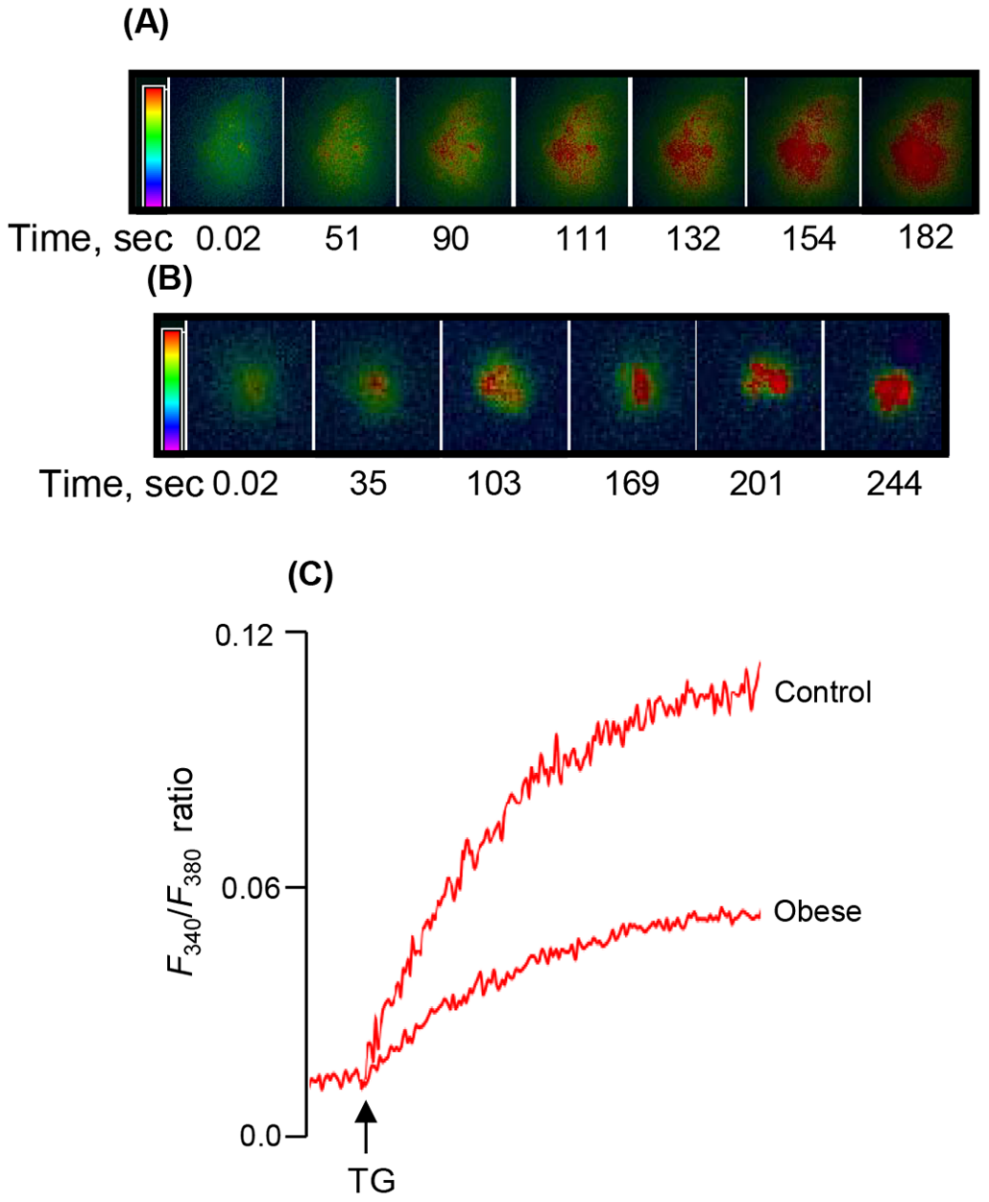
Effects of TG on the increases in [Ca^2+^]i in CD36-positive TBC. The CD36-positive TBC were isolated from CVP of *P. obesus* as described in Materials and Methods. Ca^2+^ imaging studies were performed on CD36-positive TBC in Ca^2+^-containing (100% Ca^2+^) media. The changes in intracellular Ca^2+^ (F_340_/F_380_) were monitored under the Nikon microscope (TiU) by using S-fluor 40x oil immersion objectives. Colored time-lapse changes show the kinetics of the rise in [Ca^2+^]i, induced by TG at 5 µM, in a CD36-positive taste bud cell freshly isolated from CVP of control (A) and obese (B) animals. (C) represents the single traces of observations and the arrow indicates when the test molecule (TG, 5 µM) was added into the cuvette without interruptions in the recording.

**Figure 6:**
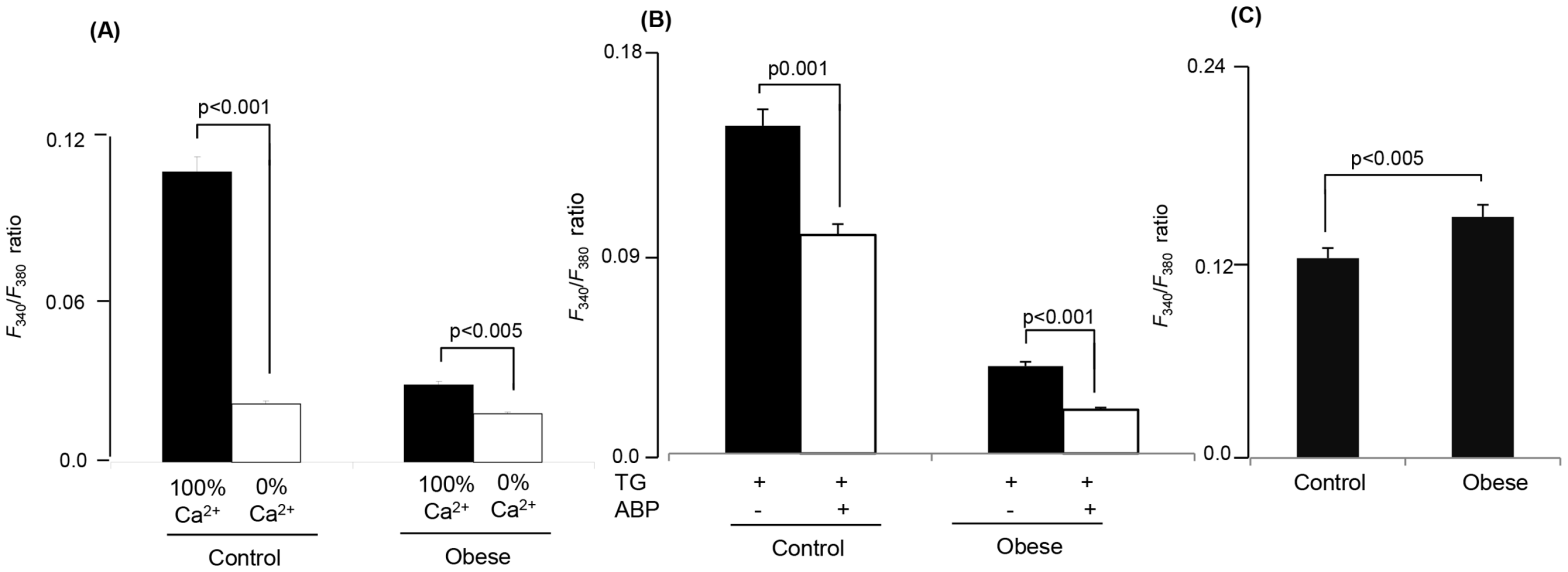
Implication of Ca^2+^ influx in TG-induced increases in [Ca^2+^]i in CD36-positive TBC. The CD36-positive TBC were isolated from CVP of *P. obesus* as described in Materials and Methods. The changes in intracellular Ca^2+^ (F_340_/F_380_) were monitored under the Nikon microscope (TiU) by using S-fluor 40x oil immersion objectives. (A) represents the histograms of the experiments performed in Ca^2+^-containing (100% Ca^2+^) and Ca^2+^-free (0% Ca^2+^) media with TG (5 µM). (B) represents the Δ increases in [Ca^2+^]i evoked by TG (5 µM) in the presence or absence of ABP at 5 µM in Ca^2+^-containing (100% Ca^2+^) medium. (C) represents the changes in intracellular Ca^2+^ (F_340_/F_380_) evoked by ionomycin at 500 nM in Ca^2+^-containing (100% Ca^2+^) medium.

In TBC, TG-induced increases in [Ca^2+^]i were lesser in obese animals than those in controls (Fig. 5A, B, C). The action of TG was curtailed in 0% Ca^2+^ buffer as compared to 100% Ca^2+^ buffer in TBC of control and obese gerbils (Fig. 6A). Addition of APB diminished the TG-induced increases in [Ca^2+^]i in TBC of control and obese animals (Fig. 6B).

Finally, we employed, ionomycin, known to open non-specifically Ca^2+^ channels [30], and we observed that the increases in [Ca^2+^]i, evoked by this agent, were not statistically different in TBC of control and obese animals (Fig. 6C).

## Discussion

*P. obesus* is a diurnal rodent that lives in the arid zones of North Africa and Eastern Mediterranean region. The “trival name” sand rat has been given to this rodent, but it belongs to the family of Muridae and subfamily of Gerbillinea. This animal does not exhibit hyperglycemia in its natural desert habitat; however, it has the tendency to develop diet-induced hyperglycemia associated with obesity. The first glance at our results confirms the previous findings that *P. obesus* subjected to the laboratory standard diet exhibit hyperinsulinemia, hyperglycemia and hypertriglyceridemia associated with an increase in body weight, compared to *Salsola foetida*-fed animals [19].

Interestingly, increased hepatic mass was positively correlated with weight gain in obese *P. obesus* [31]. We have previously shown that obesity in these animals is characterized by peripheral and hepatic insulin resistance [16]. Since excessive consumption of high caloric diet contributes to weight gain and insulin resistance, it is possible that fat intake might be altered in the obese state [32]. Besides, obese subjects have been reported to exhibit a higher preference for fatty foods than lean subjects [33]. In animal models, oral fatty acid sensitivity, in response to stimulation with fatty acids, is determinant for fat consumption and body weight regulation. We were tempted to assess the oro-sensorial detection of dietary lipids. We observed, to our great surprise, that the obese animals exhibited a strong gustatory preference for the oily solution in a two-bottle preference test. The control animals did not drink, at all, any of the liquids as they were acclimatized to feed mainly on succulent plant, *Salsola foetida*, relatively high in water and electrolytes. When we extended the taste preference experiments for two or three days or more, the obese gerbils drank oily solution so much in excess that it caused their death (not shown). The gerbils also exhibited a spontaneous preference for a sweet solution which was very high in obese group of animals. The molecular mechanisms of sweet preference and the implication of lingual receptors will be a subject of intensive investigation in the near future.

The CD36, a lipido-receptor, has been implicated in lipid taste perception in rodents [7], [8] and human beings [10], [34]. The TBC of obese animals were found to express low CD36 mRNA as compared to control animals, though the protein contents of CD36 were not altered between control and obese gerbils both in TBC and intestine (used as a control). We do not have a plausible explanation for these discrepancies. It is possible that the total CD36 protein in TBC of obese animals remains unchanged, but its distribution between lipid rafts and soluble plasma membrane fractions is altered. According to this hypothesis, the CD36 protein would be translocated to cytosolic soluble fractions, thus decreasing its contents in the lipid rafts in the TBC of obese animals. It is also possible that CD36 is degraded in ubiquitin-dependent manner in lipid rafts [35], but its contents will rise in non-raft fractions. These hypotheses should be confirmed in future. Conversely, low CD36 in the lipid rafts of TBC might contribute to low (hypo) sensitivity to fatty acids. It has been shown that the animals that are hyposensitive to orally administered fatty acids consume excess fats and develop obesity when exposed to a high-fat diet [36]. In our study, we did not measure daily food intake in obese and control animals; however, we assume that the obese animals would show a strong eating behavior toward the palatable laboratory diet because it contains 7.5% of lipids whereas *Salsola foetida* contains only 0.4% of them. It has been shown that foods high in dietary fat exert a weak effect on satiation, which leads to a form of passive overconsumption [3]. As mentioned here-before, obese *P. obesus*, in a two-bottle test, clearly showed a spontaneous preference for a solution, containing colza oil.

We have shown that LA, by binding to CD36, triggers an increase in [Ca^2+^]i in CD36-positive TBC purified from mouse CVP [23], [9]. LA induced an increase in [Ca^2+^]i in TBC *via* CD36, and it was higher in obese animals than control rodents. A plausible explanation on the increase in Ca^2+^ signaling in obese animals is not available. It is possible that other proteins like GPR120 [37], also considered as a lipido-receptor, are also participating in LA-induced Ca^2+^ signaling as SSO [28], the CD36 blocker, significantly curtailed, but did not completely abolish, the increases in increase in [Ca^2+^]i in these cells. However, the implication of GPR120 in Ca^2+^ singling in these cells remains to be assessed later on. We tried to detect GPR120 in the TBC of *P. obesus* in western blots, by using antibodies form Abcam (ab-75313) and Santacruz (sc-48203), but without success, and it may be due to species different. In future, we will employ anti-GPR120 antibodies from other sources. Alternatively, it is also possible that altered composition of plasma membrane phospholipids of CD36-positive TBC in obese animals might be responsible for altered downstream signaling, involving the hydrolysis of phosphatidylinositols, but this hypothesis remains to be ascertained in future.

However, a decrease in LA-induced Ca^2+^ signaling in 0% Ca^2+^ medium in TBC of control and obese animals show that this fatty acid evokes increases in [Ca^2+^]i from intracellular pool, probably from endoplasmic reticulum (ER) as demonstrated in mouse CD36-positive TBC [9], [24]. As per capacitative model of Ca^2+^ homeostasis, the depletion of intracellular stores of Ca^2+^ is followed by Ca^2+^ influx *via* the opening of Ca^2+^ channels to refill the intracellular pools [38]. These channels have been termed store-operated Ca^2+^ (SOC) channels, and their activity is maintained as long as the stores are not refilled [38]. To determine whether LA-induced recruitment of Ca^2+^ from ER results in the opening of SOC channels, we employed a SOC channel blocker, which as anticipated, decreased significantly the LA-induced calcium response in 100% Ca^2+^ buffer in TBC of control and obese animals. In order to probe the implication of ER pool, we employed SERCA inhibitor and observed that TG-induced increases in [Ca^2+^]i in TBC of obese animals were lower than those in controls. These observations confirm the above-mentioned results on LA-induced decreases in [Ca^2+^]i in 0% Ca^2+^ buffer in TBC of obese animals, indicating a decreased recruitment of Ca^2+^ from the ER pool. As expected, TG also opened SOC channels both in control and obese TBC. The modification of opening of SOC channel is a specific physiological feature in these animals, as the action of ionomycin, known to open non-specifically almost all the Ca^2+^ channels, was not significantly different between TBC of control and obese *P. obesus*.

As regards *in vivo* calcium homeostasis, these animals possess regulatory adaptive mechanisms. It has been shown that plants of chenopodiacae family provide with much oxalate (300 mg/d), but little Ca^2+^ (30 mg/day); whereas the laboratory diet provides with low-oxalate (<100 mg/day) and high-Ca^2+^ (approximately 150 mg/day). *P. obesus* has the ability to eliminate dietary oxalate and regulate Ca^2+^ homeostasis *via* intestinal symbiotic bacteria whose number increases or decreases according to the diets [39].

In conclusion, we report in this study, for the first time, that obesity in *P. obesus* is associated with increased oro-gustatory perception of dietary fat and upregulated Ca^2+^ signaling in circumvallate papillae, associated with altered CD36 expression. Future challenges require in-depth investigations to better understand the implication of GPR120 or the downregulation/distribution of CD36 in plasma membrane fractions of TBC during obesity since the mechanisms responsible for altered Ca^2+^ signaling might be involved in the regulation of feeding behavior particularly post-prandial phase of food ingestion.
